# Smartphone-Based Colorimetric Analysis of Urine Test Strips for At-Home Prenatal Care

**DOI:** 10.1109/JTEHM.2022.3179147

**Published:** 2022-05-30

**Authors:** Madeleine Flaucher, Michael Nissen, Katharina M. Jaeger, Adriana Titzmann, Constanza Pontones, Hanna Huebner, Peter A. Fasching, Matthias W. Beckmann, Stefan Gradl, Bjoern M. Eskofier

**Affiliations:** Department of Artificial Intelligence in Biomedical EngineeringFriedrich-Alexander-Universität Erlangen–Nürnberg 91052 Erlangen Germany; Department of Gynecology and ObstetricsUniversitätsklinikum Erlangen 91054 Erlangen Germany

**Keywords:** Urinalysis, smartphone, colorimetric analysis, artificial intelligence, digital health

## Abstract

Objective: Clinical urine tests are a key component of prenatal care. As of now, urine test strips are evaluated through a time consuming, often error-prone and operator-dependent visual color comparison of test strips and reference cards by medical staff. Methods and procedures: This work presents an automated pipeline for urinalysis with urine test strips using smartphone camera images in home environments, combining several image processing and color combination techniques. Our approach is applicable to off-the-shelf test strips in home conditions with no additional hardware required. For development and evaluation of our pipeline we collected image data from two sources: i) A user study (26 participants, 150 images) and ii) a lab study (135 images). Results: We trained a region-based convolutional neural network that is able to detect the urine test strip location and orientation in images with a wide variety of light conditions, backgrounds and perspectives with an accuracy of 85.5%. The reference card can be robustly detected through a feature matching approach in 98.6% of the images. Color comparison by Hue channel (0.81 F1-Score), Matching factor (0.80 F1-Score) and Euclidean distance (0.70 F1-Score) were evaluated to determine the urinalysis results. Conclusion: We show that an automated smartphone-based colorimetric analysis of urine test strips in a home environment is feasible. It facilitates examinations and provides the possibility to shift care into an at-home environment. Clinical impact: The findings demonstrate that routine urine examinations can be transferred into the home environment using a smartphone. Simultaneously, human error is avoided, accuracy is increased and medical staff is relieved.

## I. Introduction

Urinalysis with test strips is a common, simple to use and routinely applied screening method during prenatal care for the early detection of pregnancy-related diseases [1]. During prenatal care, standard urinalysis test strips are frequently applied. Regular screening throughout pregnancy every two to four weeks is recommended to detect urinary tract infections, proteinuria, glucosuria or asymptomatic bacteriuria at an early stage [1], [2].

The test strip is briefly dipped into a urine sample from the patient to completely moisten all indicator fields. The subsequent discoloration of the indicator fields is then usually evaluated visually against a reference card by medical personnel. Even though the visual evaluation is time-consuming and error-prone, it is often not used in practice as the financial costs of automated analysing devices are too high [3], [4]. Furthermore, test results are frequently not documented, especially negative results, which makes it impossible to reproduce the patient’s history at a later point in time. Integrating smartphones into the evaluation of printed diagnostic tools such as urine test strips could facilitate examinations and improve quality of care by eliminating observer-related errors. Patients could benefit from a shift to the at-home environment by fewer on- site appointments.

Our key contribution is the development of a method to differentiate and automatically measure 10 different urinalysis parameters from images captured with a smartphone in a home environment.

## II. Related Work

An automated evaluation of urine test strips that using smartphones has already been investigated in previous work. A large number of these studies focused on improving accuracy and reproducibility by employing custom build equipment like custom-built opaque boxes or optical attachments to the camera were used [5]–[9].

While these studies used commercially available test strips, others employed custom-made test strips to facilitate the evaluation through a smartphone [10], [11]. Han *et al.* developed urine test strips where the reference fields are located directly around the test fields. Apart from that Anthimopoulos *et al.* used self-developed color reference cards to enable a reproducible color correction [12]. The smartphone-based urinalysis for commercially available urine test strips is also possible without any further equipment [13]–[15].

Previous studies used different approaches to automatically detect the urine stick in the captured images, such as an optical marker attached to the stick to recognize the position and orientation [16]. Hong and Chang developed a template matching algorithm, that uses a mono-color template with the periodic pattern of test strip with its single fields [14]. The algorithm then estimates pixel-by-pixel the most probable position. In the work of Wang *et al.* a Laplacian edge detection was applied to a certain area in the image, as the position of the urine stick in the image was predefined during the image capturing process [17]. The ORB detector that is also used in this work was previously already applied to detect urine sticks and color reference cards [12]. A high degree of accuracy with simultaneously advantageous computational efficiency was achieved. The existing research used a variety of approaches to analyse and compare colors. The matching factor, one of the methods applied in this work, has already been used with good results. It incorporates the weighted values of the individual color channels in the HSV color space [18]. Fletcher *et al.* incorporated a weighted k-nearest neighbors algorithm and classifier, while the approach of He *et al.* was to use a quadratic discriminant analysis [13], [16]. Three systems were identified that already underwent an approval process as a medical device. One of them is the Uchek urinalysis device of Biosense Technologies [19]. The study showed, such a system can increase the efficiency of health personnel and reduce the risk of human errors that often occur when test strips are visually read.

Furthermore, the feasibility and acceptability of a smartphone-based urinalysis system within prenatal care have been analyzed [20]. The authors concluded that the smartphone-based test strip testing is overall well accepted and that their participants preferred self-testing. As a third system Scanwell health developed a test kit for urinary tract infections that detects leukocytes and nitrite in the urine through a smartphone application and self developed test strips [21]. All three systems have in common that they require additional hardware or self-developed test strips.

The reviewed works on smartphone-based colorimetric analysis of urine test strips have demonstrated high accuracy in a laboratory setting. However, there has been no investigation of such a method’s performance in a home-based environment. Therefore, the purpose of this work is to develop and evaluate a smartphone-based method for an automated analysis of commercially available urine test strips that is usable in a home environment by non-trained persons and the integration in a digital pregnancy care concept.

## III. Methods

**FIGURE 1.**
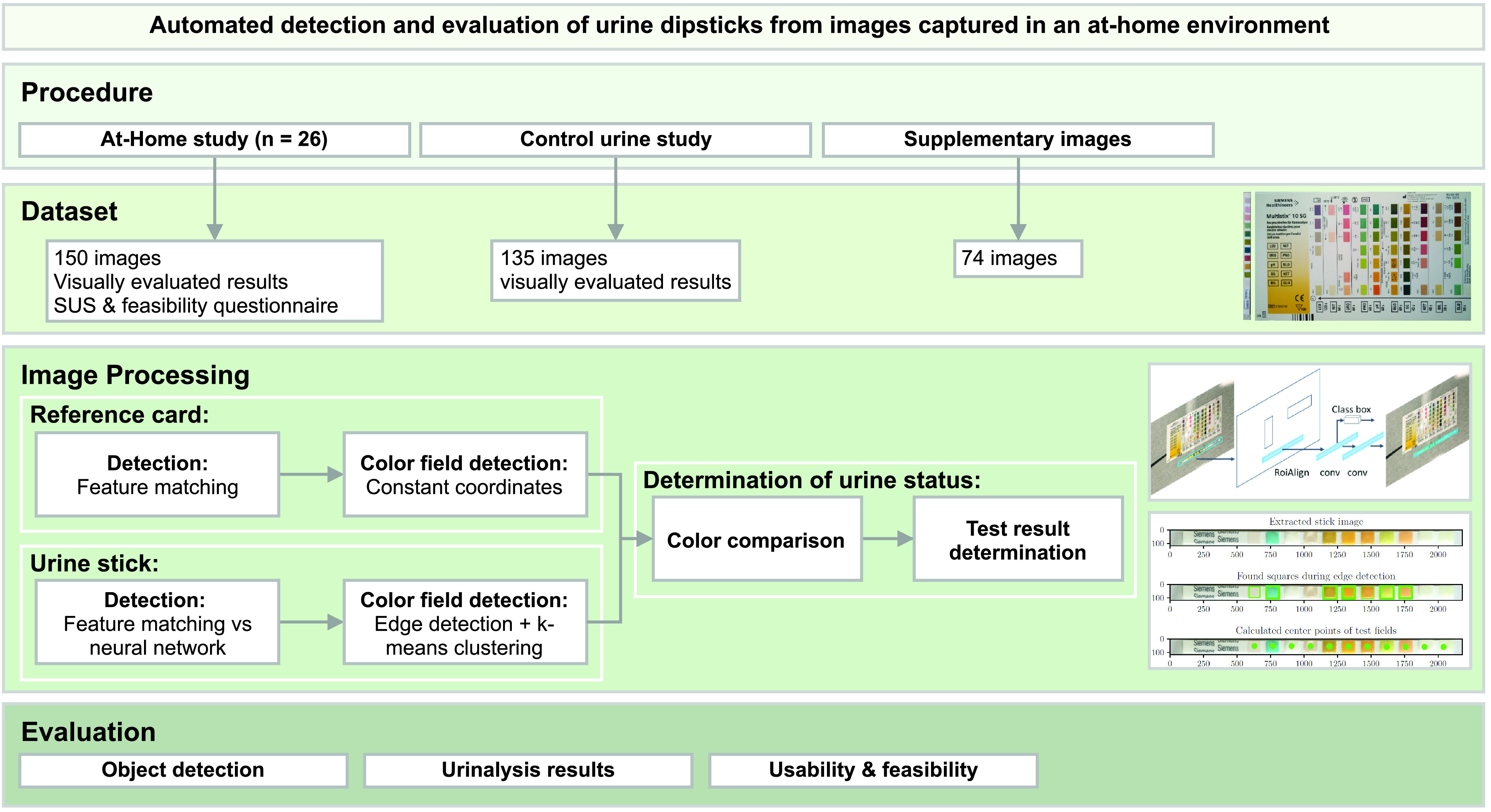
Overview diagram describing the pipeline of the automated detection and evaluation of urine test strips, including the conducted studies, the resulting dataset and further image processing.

**FIGURE 2.**
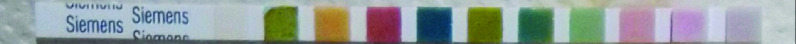
Standard urinalysis test strip with 10 test fields after reaction with control urine.

For an automated colorimetric analysis of the urine sticks, the position of the urine stick and the reference card in the image is first determined. Afterwards the location of the single test fields of the urine stick and the reference fields of the card need to be detected. As soon as these positions are known, the color of each field can be analyzed and compared. We conducted two studies in order to develop and validate our pipeline. An overview of the overall process of this work is shown in Figure 1. For the urine examination, we employ the Multistix 10 SG test strips by Siemens Healthineers AG (Erlangen, Germany) including the following 10 parameters: glucose, bilirubin, ketone, specific gravity, hemoglobin, pH value, protein, urobilinogen, nitrite and leukocytes. An exemplary urine test strip of the ones used in this work is shown in Figure 2.

### A. At-Home Study

#### 1) Participants

In total 26 participants (34.0±11.6 years, half male, half female, diverse educational background) conducted the urine examination at home with a test kit, containing two test strips, a reference card and a plastic cup. In total 23 different devices were used for study conduction, both Android and iOS systems. The study was conducted after approval of the ethics committee of the Friedrich-Alexander University Erlangen-Nürnberg (106_13 B).

#### 2) Web-Application

**FIGURE 3.**
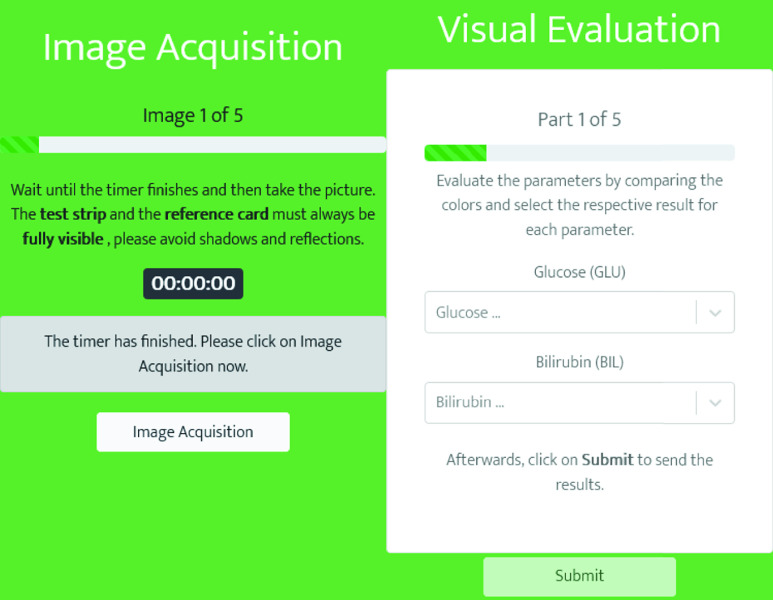
Web-app interface for image acquisition (left) and documentation of visually evaluated results (right) provided within the at-home study.

In order to facilitate image acquisition, test result documentation and time compliance for untrained study participants, a web application was provided. This application guided users through each step and was implemented as progressive web app. As such, it is operating system independent, requires no app marketplace distribution or user installation. Sample screenshots of this web application from two steps are shown in Figure 3. ReactJS served as foundation for the application front end [22]. This front end is connected to a NodeJS backend to store the incoming data [23].

#### 3) Procedure

**FIGURE 4.**
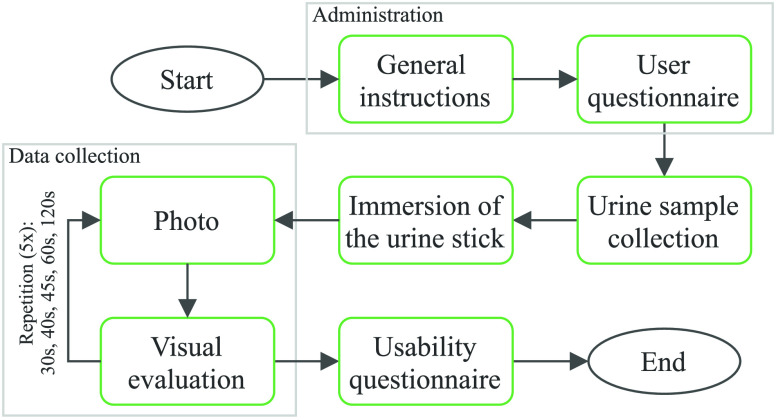
Workflow of the urine examination with the web-app for all study participants.

The procedure for the user conducting the study is schematically illustrated in Figure 4. Initially, general instructions are provided in order to ensure an adequate preparation. Subsequently, the user is instructed to collect the urine into a dry and clean cup. After confirming an explanation is presented, how the test strips should be dipped into the urine. In the following step, participants were told to place the stick on an even and dry surface next to the reference card. Once it has been confirmed for the test strip to be immersed, a timestamp is saved and a timer is started in the background.

The reaction of the test fields with the urine is a time-dependent reaction and therefore the manufacturer defines specific time points for each parameter to be evaluated, after 30, 40, 45, 60 and 120 seconds. A timer ensures, that parameters are evaluated after each time point.

In order to evaluate the feasibility of self-testing and the usability of the provided guidance app, the participants filled out a questionnaire including the System Usability Scale (SUS) as well as custom questions aiming to assess how well the participants coped with the urine test strips, the visual evaluation and the time specifications [24], [25].

### B. Control Urine Study

Since no pathological measurements were expected in healthy participants in the home environment study, an additional study was conducted with control urine manufactured by LT-SYS (Berlin, Germany). This reagent, as it is made from human urine, has to be considered to be potentially infectious. Therefore, a handling by non-trained participants at home was not feasible. Control urine is available in two levels, one that guarantees measured values simulating a healthy patient, while level two delivers measurements in a certain pathological range.

All ten test fields of one urine strip were moistened and a timer was started manually. After the times specified by the manufacturer of the urine test strips, both an image was taken and the measured values were read visually. This procedure was repeated with 12 different smartphones.

The image acquisition was performed with the built-in camera applications of the smartphones. Different randomly chosen backgrounds, light conditions and image angles were chosen to achieve a high variance of images. Most of the devices were used at least twice with different light conditions (natural, artificial) generating 27 sets of five images each. Five devices operated on Apple iOS, five on Google Android and two on Windows Mobile.

### C. Dataset

Our experiments resulted in a total of 285 images: 150 from the at-home study and 135 from the control urine study. To improve the development process of the object detection pipeline, 74 supplementary images that were excluded for the colorimetric analysis were captured. They contain especially strongly patterned and unusual backgrounds with randomly chosen other objects, very dark or bright lighting conditions and unusual imaging angles of the urine sticks.

### D. Image Processing

#### 1) Object Detection

In every image, a test strip and its respective reference card need to be identified before the test results can be determined. Prior to the presented pipeline in this work, we evaluated several different methods for the application at hand. Most promising were feature matching and a region-based convolutional neural network. Feature matching was chosen due to its simplicity, efficiency and potential adaptability to test strips of other manufacturers.

##### a: Data Labeling

As a preparation in all images the urine sticks were labelled with the open source VGG Image Annotator [26]. The extracted annotations represent the urine stick as a set of polygon points.

##### b: Feature Matching

**FIGURE 5.**
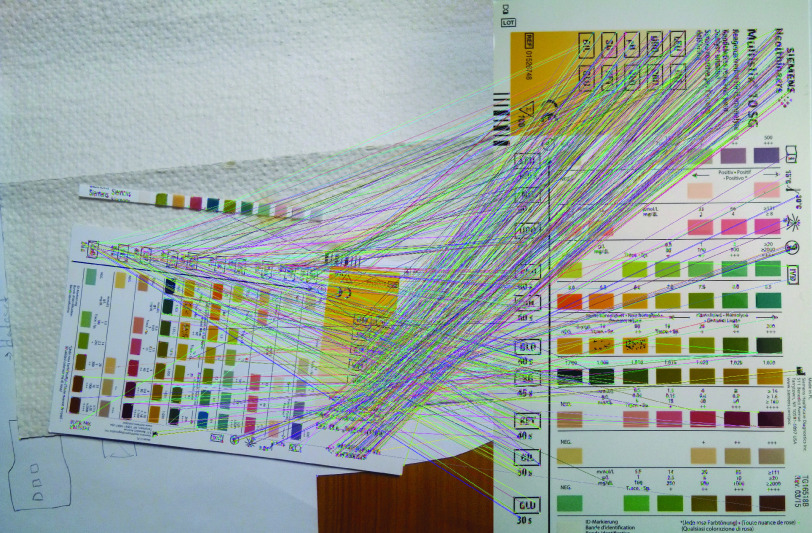
Found matches of the feature matching algorithm in an example image.

The chosen method for the feature detection and matching is based on the ORB feature detector implemented with the open-source library OpenCV [27], [28]. The basic workflow is identical for both objects. The images that will be compared are converted to grayscale and the orb detector searches and calculates keypoints and descriptors in both images. A brute force feature matching is applied to the detected descriptors. According to their Hamming distance all matches are sorted and only best matches are kept for further processing. An example of those found matches is shown in Figure 5. A homographic warp is calculated to map the points of the source image to the desired destination as specified in the input reference. The homography matrix $H$ indicates the perspective transformation of the source points to the destination by minimizing the back projection error.

##### c: Region-Based Convolutional Neural Network

As a second approach for the detection a region-based convolutional neural network (R-CNN) was chosen. The used implementation of Mask R-CNN is based on the existing implementation of Matterport Inc. (Sunnyvale, US), which uses the open-source libraries Keras and Tensorflow [29]. It is based on a Feature Pyramid Network (FPN) and a ResNet101. This implementation of a region-based convolutional neural network (Mask R-CNN) was applied in previous works for image segmentation tasks [30], [31]. These showed that comparably small training sets sizes were sufficient to reach a high accuracy. We used transfer learning with the model already pre-trained on the MS COCO dataset [32].

Training and testing was performed using a three-fold cross validation. In order to avoid similar images appearing in both the test and training set, the images are grouped subject-wise and respectively experiment-wise for the control urine. In total the training was performed for 15 epochs with 200 steps per epoch and a learning rate of 0.001. The steps per epoch were chosen according to the small size of the training set to ensure every image is processed during one epoch. Training of the neural network was performed until convergence.

##### d: Comparison

The coordinates of the corners of the stick within the original image were calculated for both methods. These coordinates are then compared to the annotations containing the ground truth position of the urine stick. Only if the rotated bounding rectangle is within the ground truth the detection was classified as successful. Since the ground truth image of the urine stick that is used for the feature matching has a border around it, a tolerance of 10 percent similar to this border is added to the found coordinates of the Mask R-CNN. This ensures an equal comparison of both methods.

#### 2) Reference Field Detection

As the detection of the reference card is working accurately, the single reference fields can be extracted through constant pixel coordinates from the extracted reference card. Therefore distances were measured in the reference image of the card. These values are used to find the single fields in the images of the detected card. In order to ensure the correct extraction, the separated color fields were reviewed visually.

#### 3) Test Field Detection

Since the extracted urine sticks from the images are not as uniform as the extracted reference cards, measuring coordinates from a reference image is not sufficient. Therefore, in order to determine the positions of the test fields automatically different approaches were chosen. As a first approach, histograms were calculated from the saturation channel after converting the image to the HSV color space. Through a Nelder-Mead optimization the test fields were detected [33]. However, this approach was not able to accurately and reliably detect the positions of the single fields, especially with dark backgrounds, and a skewed image recording perspective.

As a second approach a custom pipeline based on an edge detection was implemented. First, the image of the urine stick is preprocessed several times with different filters, blur and threshold settings and an edge detection is performed with each setting. Each time the detected squares with a minimum area of 20% of the total image height and an aspect ratio of 1 ± 0.06 are saved. In the end all found squares are used to cluster the center points. This clustering is implemented through a k-means algorithm [34]. Since not all of the test fields are always detected, the number of clusters is determined by the Elbow method [35]. Afterwards the determined center points are analyzed and if distances exceed the median distance of more than 50% depending on the given points, or the number of clusters does not match the number of test fields, missing points are added automatically.

#### 4) Color Comparison

Three different approaches were used to compare the colors of the test fields with their reference fields: Hue value comparison, Matching Factor and Euclidean distance. All approaches rely on a color transformation of the image sections of the detected test and reference fields into the HSV color space. This color space represents colors similar to the human perception and colors perceived similar by the human, are also adjacent in the HSV color space [36]. The hue channel describes the dominant wavelength of the present color, therefore this channel contains the most relevant information to analyze and compare the colors. For all three approaches the values for each color channel were determined through the peak value in the respective histogram.

Due to these characteristics of the color space, the first approach for the comparison of two colors is to compare the hue channels, as shown in Equation 1.

\begin{equation*} Similarity_{Hue}= 1 - \frac { \Delta H }{H_{max}} \tag{1}\end{equation*}

For the test field and its related reference field, the histogram of the hue channel is generated with the built-in OpenCV function and the peak value is determined. $\Delta H$ represents the difference of the peak values. Equation 2 shows the applied formula to calculate the matching factor in the HSV color space according to [18]. For all three channels the histograms are generated and the peak value is determined. With $\Delta H, \Delta S$ and $\Delta V$ representing the difference for each channel of the test field and its related reference fields. Additionally, weight factors are introduced, with $\alpha = 0.6429 $ and $\beta = 0.1786 $ analogous to [18].

\begin{equation*} MF_{HSV}= 1 - \frac {\alpha \Delta H + \beta \Delta S + \beta \Delta V}{H_{max} + S_{max} + V_{max}} \tag{2}\end{equation*}

The values of each channel of the HSV color space can be interpreted as cylinder coordinates. With these coordinates the Euclidean distance between two points and therefore two colors can be calculated as shown in Equation 3.

\begin{equation*} Distance_{HSV}= 1 - \frac { \sqrt {\Delta x^{2} + \Delta y^{2} + \Delta z^{2}}}{\sqrt {(2\,S_{max})^{2} + V_{max}^{2}}} \tag{3}\end{equation*}

#### 5) Test Result Determination

For all three color comparison methods, the color field representing the highest similarity calculated with each method defines the test result for the respective parameter. Since all three approaches are based solely on the values of the color channels, they are purely deterministic, not relying on training data. The calculated test result is compared with the visually determined result documented during the study conduction. For all parameters the test results are classified in terms of a confusion matrix. In the urine of a healthy patient glucose, bilirubin, ketone, blood, protein, nitrite and leukocytes are below the detection limits of the urine sticks. The pH value is pathological above a value of 7.0. Specific gravity is classified as negative between 1.005 g/ml and 1.025 g/ml [37], [38]. The concentration of urobilinogen is classified as negative for a concentration below 1 mg/dl [38].

## IV. Results

### A. Object Detection

As listed in Table 1, the feature matching algorithm detected 40.7% of the urine sticks correctly. In contrast, the Mask R-CNN was able to detect 85.5% of the sticks correctly. Of all reference cards 98.6% were correctly detected. Three images from the at-home study were excluded from further processing because the reference card was not fully depicted and thus several reference fields were missing.

**TABLE 1:** Object Detection Results of the Urine Sticks for Two Different Approaches

Method	Number of test images	Successfully detected	Not detected
Feature Matching	150 (100%)	61 (40.7%)	89 (59.3%)
Mask R-CNN Model	117 (100%)	100 (85.5%)	17 (14.5%)

### B. Color Comparison and Test Result Determination

**FIGURE 6.**
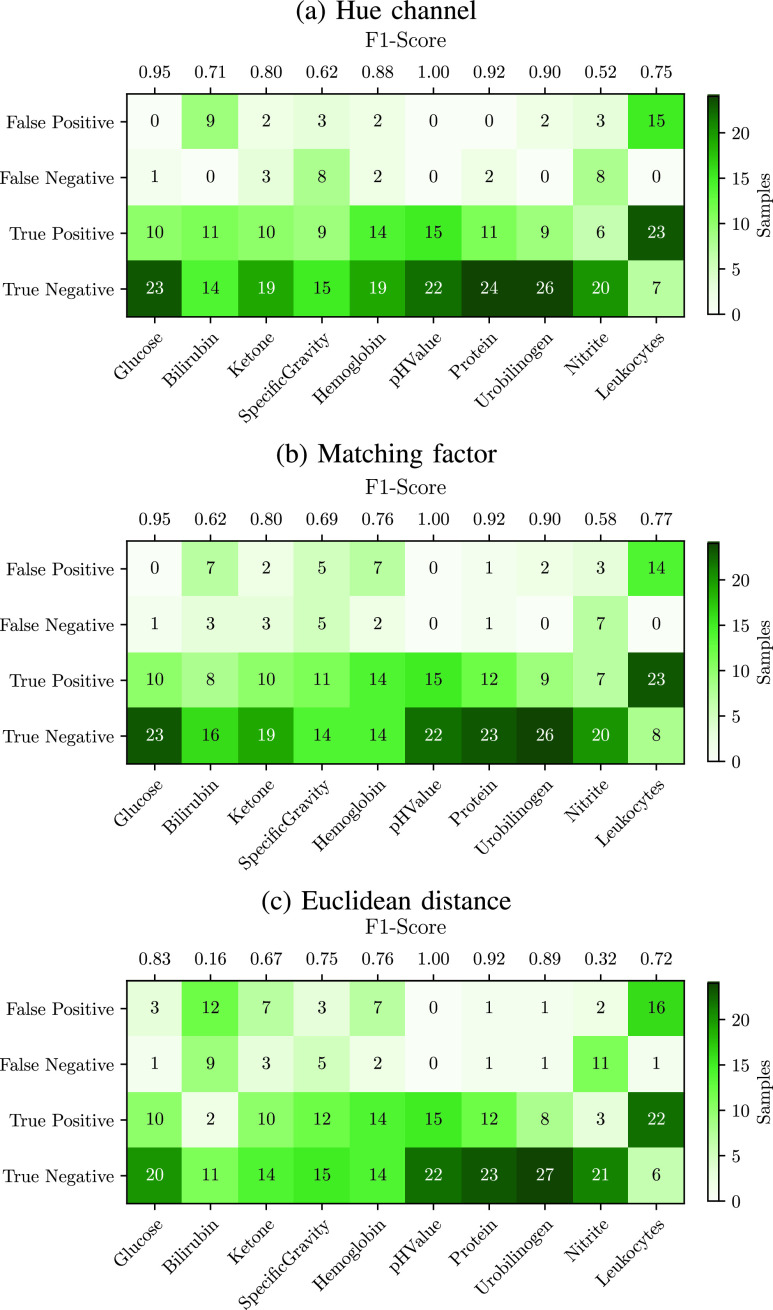
Confusion matrices of the automated urine test strip evaluation using three different methods: a) comparison of hue values, b) matching factor, c) Euclidean distance.

Figure 6a shows a confusion matrix for all parameters and images generated during both studies. As the matrix representing the results for the similarity of hue values in Figure 6a illustrates, the majority of test results was classified correctly as either true negative or true positive. This is similar to the test results calculated through the matching factor in Figure 6b as well as the euclidean distance, shown in Figure 6c. All three methods were able to classify all test results of pH-values correctly. In contrast, a high rate of false positive classifications is generated by all three methods for the leukocyte test result. The euclidean distance reached the lowest average F1-score of 0.70, the matching factor 0.80. The highest accuracy was reached through the comparison of hue values, with an average F1-score of 0.81.

### C. Feasibility and Usability

**FIGURE 7.**
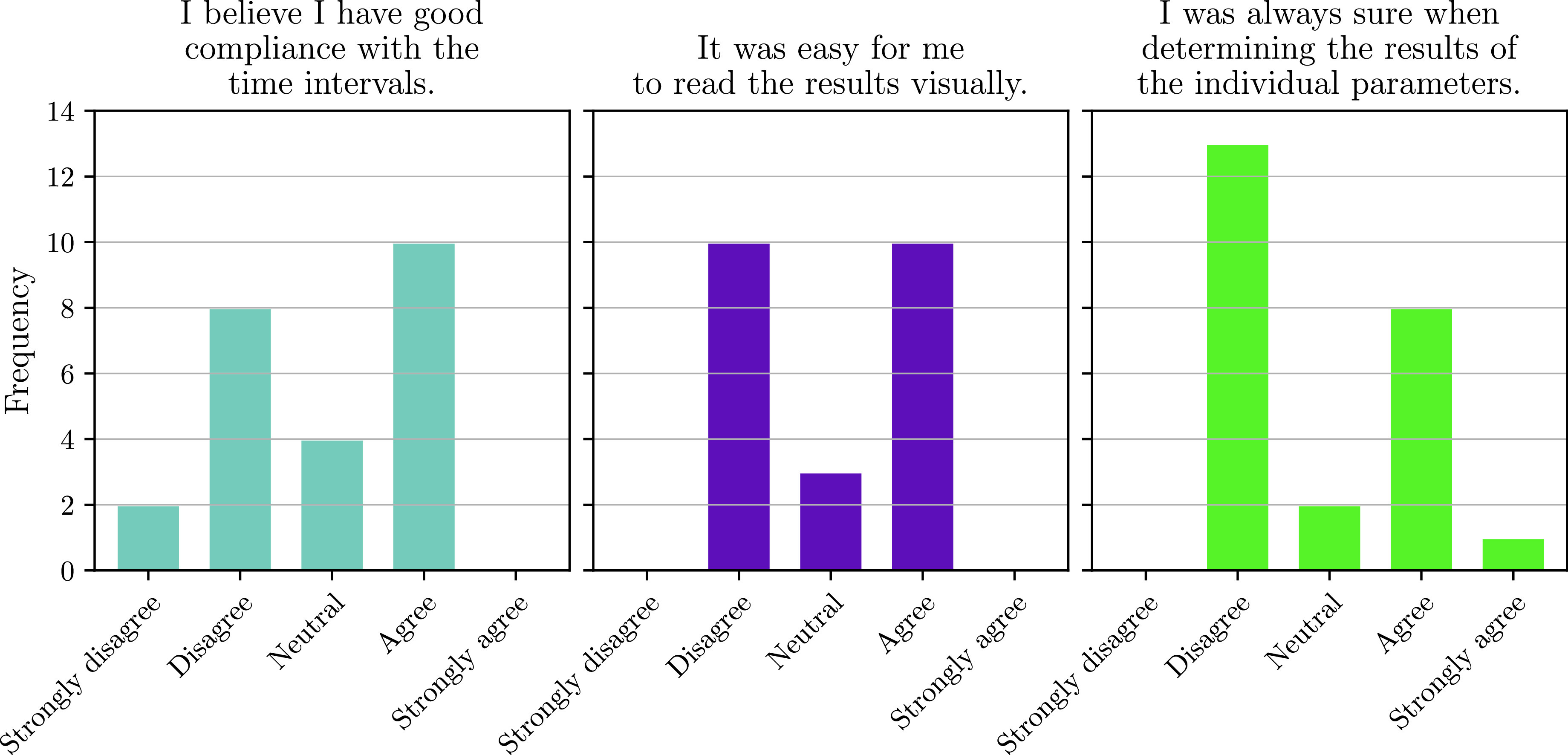
Distribution of answers to the target questions from participants in the at-home study.

24 participants answered the target questions regarding the feasibility. The results to those questions is shown in Figure 7. As the answers indicate, insecurity regarding the time required for the conduction was present among the participants. Furthermore, insecurity arose during the determination of the test results. The answers to the usability questionnaire resulted in an average SUS score of 71.9±17.3.

**FIGURE 8.**
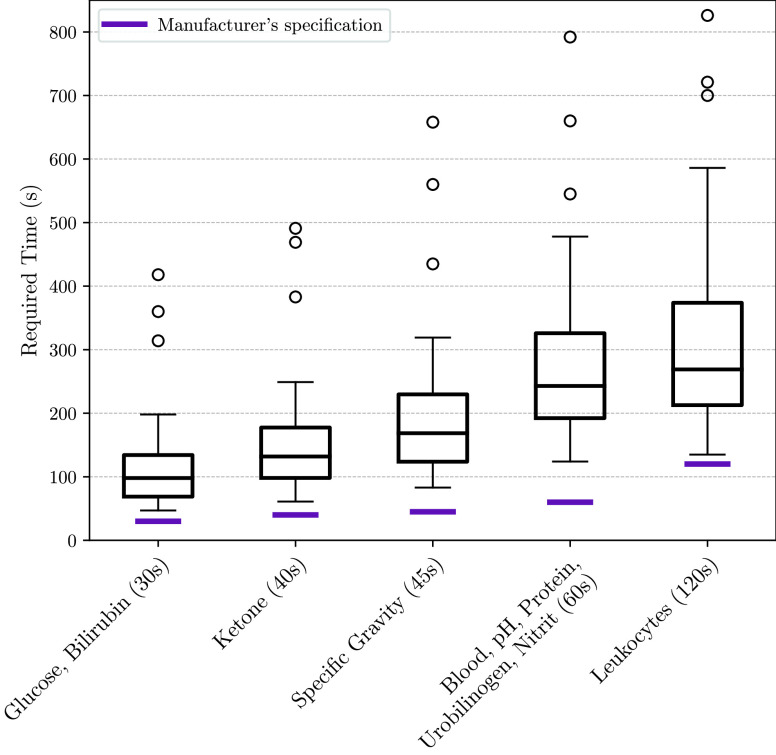
Required time for conducting the urine analysis of the study participants.

Figure 8 shows the duration the participants required for the completion of a urine examination including the image acquisition and documentation of visually read results. In purple marked is the manufacturers specification. The boxplot highlights that there was an augmented demand for time and parameters could not be evaluated within the specifications. Especially the fourth time step, where five different parameters need to be compared shows significant deviations.

## V. Discussion

Within this work a complete pipeline was implemented to detect and evaluate urine test strips automatically from images captured with a smartphone. Two studies were conducted to generate images and evaluate the accuracy of the chosen methods.

During the at-home study insecurity regarding the visual determination of test results was present among the participants. This was indicated in both the feasibility and usability questionnaires. A correlation with the level of education could not be observed. The reason for this can be a missing training prior to the conduction of the study. Most participants only conducted the study once. All participants received two test strips and many of them required both, because in a first trial often the orientation of the stick in relation to the reference card was unclear. The conduction was then aborted and restarted with the second stick. Those participants verbally indicated a higher confidence during the conduction in the second attempt.

Furthermore, the distances between the single test fields do not match to those of the related fields on the reference card. This increases the difficulty to assign the fields correctly especially on time. Due to the insecurity during the visual evaluation, test results that are used as ground truth may be incorrectly documented, which could have a negative impact on performance. Especially results for the classification of leukocytes show a significant difference between images obtained during the at-home study and the control urine study. While test results of the control urine study were classified correctly for more than 60% only 21% of the at-home study were classified correctly. Significant differences between the devices could not be identified due to the small number of smartphones. Similarly, the sample size was too small for a statistical analysis of the influence of the different levels of education of the participants.

An important limitation regarding both studies is the coverage of measured values. Since the recruited participants are mostly healthy and the used control urine is only available in two specific levels, the range of possible measured values could only be covered partially. However, the focus of this work was to investigate whether an automated evaluation of urine test strips in an at-home setting is feasible and whether the determined values correspond to the test results of the participants.

The majority of users was not able to conduct the urine examination within the time specified by the manufacturer. Although this means that the urine status of the participants was not validly determined, however through the parallel visual evaluation and image acquisition the color comparison and hence the values used as ground truth are still valid.

The presented pipeline is implemented to perform an automatic detection and evaluation of urine test strips. The single steps, the detection, the field extraction and the colorimetric analysis can be connected in series without a manual operation in between. The majority of test results calculated from images obtained during both studies were classified correctly. Certainly, the reached accuracy, especially for the color comparison methods is debatable for a valid and reliable urine status determination.

The fully automated detection of both urine sticks and reference cards as well as the extraction of the single color fields as implemented appears promising. The chosen feature matching approach was able to robustly and accurately detect the reference cards from images with a wide variety of perspectives, backgrounds and light conditions. Only for few images, a detection was unsuccessful. The reason for this is most probably a strong blur in the respective images. The feature detection is dependent on generally sharp images. In order to address this problem, further preprocessing and parameter optimization of the detection can improve the performance. Alternatively, blurry images could be directly intercepted after capturing, so users would have to capture the image again.

As pointed out, a feature matching based detection of urine sticks is not promising. The number of successfully extracted sticks of 40% is too low to be further prosecuted. As the generally small amount of detected features indicates, a further optimization of parameters and preprocessing steps would not be able to significantly improve the output of this approach. In contrast to this, the detection of urine sticks with a region based convolutional neural network appears promising. The used pre-trained model was able to reach an accuracy of 0.82 with a comparably small training set of 117 images. There is a high potential to improve the implemented method through an extension of the dataset and further parameter tuning.

When focusing on the parameters glucose and protein, which are especially relevant during prenatal care, all three methods demonstrate a high sensitivity of 0.90 and a specificity between 0.87 and 1.00 for glucose. Nevertheless, the number of samples evaluated is comparatively small and not all possible values for the individual parameters occur in the dataset. On average the F1-score of all three methods lies between 0.70 for the color comparison through the euclidean distance and 0.81 for the comparison through the hue values.

Accordingly, none of the chosen methods for the color comparison of all the parameters on its own is able to validly determine the urinalysis results. A combination of those methods may be able to already improve the accuracy. Still, further approaches need to be analyzed, especially for those parameters with an insufficient accuracy.

According to [39] a system with a SUS score above 70 can be considered good, but needs further improvement. Accordingly, the average score of 71 is above this threshold. For future studies and applications this score could be increased either by improving the web-application or by employing easier-to-use test strips.

## VI. Conclusion

The digitalisation of examinations and treatments offers the possibility to enable a more efficient, targeted and patient-friendly medical care. Patients and medical personnel invest a lot of time for routine controls that could be easily shifted into a home environment. Against the backdrop of a global pandemic, home monitoring offers a simple and safe way to assure patient health without compromising the quality of care. The application is not only conceivable in the context of prenatal care; other medical areas could benefit equally.

In this work, we presented a pipeline that is applicable in real world conditions and can be adapted to generic urine test strips. This can improve care in the future and ensure broad adaptability to different care modalities.

The implemented pipeline is a solid foundation for further research and development. Within the scope of further studies, especially the integration of a more accurate ground truth is essential. Only with a reliable ground truth a complete validation of the implemented pipeline is possible. For this purpose, a color analysis system for urine test strips can be used. However, the application of such a device in a larger study aiming to depict the home environment is not feasible. Therefore, the accuracy of visually evaluated results as ground truth can be improved through the incorporation of urine test strips with a better usability. Furthermore, the investigation of the influence of sensing parameters of the smartphones have the potential to further increase the accuracy of the presented pipeline.

Future work should include a larger number of study participants to promote a higher variation of measurement values. In addition, a test application in antenatal care will allow a better assessment of the user needs of the target group and help identifying key factors of usability for a user-centered and accurate evaluation of urine tests in the home setting. For this purpose, we plan to evaluate and further develop our algorithm in a larger study with pregnant women in the near future. A final goal is to make the results available to the users on their smartphones in real time.
